# Modeling brain functional connectivity at rest

**DOI:** 10.1186/1471-2202-14-S1-P171

**Published:** 2013-07-08

**Authors:** Vesna Vuksanović, Philipp Hövel

**Affiliations:** 1 Institute for Theoretical Physics, Technical University, Hardenbergstr. 36, 10623 Berlin, Germany; 2 Bernstein Center for Computational Neuroscience, Humboldt University, Philippstr. 13, 10115 Berlin, Germany

## 

Well organized spatio-temporal low-frequency fluctuations (< 0.1 Hz), observed in blood-oxygen-level-dependent (BOLD) signal during rest, have been used to map several consistent resting state networks (RSNs) in the brain [1-3]. It has been hypothesized that these correlated fluctuations reflect synchronized variations in neural activity of particular brain areas, which are dynamically coupled to one another forming functional connections within networks of brain. Furthermore, it has been suggested that resting state functional connectivity (FC) is strongly shaped by underlying anatomical connectivity (AC). However, although RSNs reflect anatomical connections between brain areas comprising the networks in focus, FC cannot be understood in those terms alone [4]. Here, we combine experimental and modeling approach to investigate dynamics underlying correlated behavior of distant cortical regions and formation of the so called functional networks. We aim to address complicated interplay between network structure, dynamics of its components and emerging global behavior, as key ingredients of the networks complexity [5]. We study how functional connectivity arise from anatomical connections and compare obtained data with the networks simulated on the empirically derived FC networks from resting state fMRI data. We compare two distinctive networks: one with 90 brain regions defined using the Automated Anatomical Labeling (AAL) template [6], and another with 100 regions organized into seven distinctive resting state functional networks [7]. We choose to model local network dynamics by excitable FitzHugh-Nagumo oscillators subject to uncorrelated white Gaussian noise and time-delayed interactions to account for the finite speed of the signal propagation along the axons. We discuss FC between brain regions without apparent anatomical connections, exploring dynamics that underlie these correlations.
